# Numerical Study of Cattaneo-Christov Heat Flux Model for Viscoelastic Flow Due to an Exponentially Stretching Surface

**DOI:** 10.1371/journal.pone.0137363

**Published:** 2015-09-01

**Authors:** Junaid Ahmad Khan, M. Mustafa, T. Hayat, A. Alsaedi

**Affiliations:** 1 Research Centre for Modeling and Simulation (RCMS), National University of Sciences and Technology (NUST), Islamabad, 44000, Pakistan; 2 School of Natural Sciences (SNS), National University of Sciences and Technology (NUST), Islamabad, 44000, Pakistan; 3 Department of Mathematics, Quaid-I-Azam University 45320, Islamabad, 44000, Pakistan; 4 Nonlinear Analysis and Applied Mathematics (NAAM) Research Group, King Abdulaziz University, P. O. Box 80257, Jeddah, 21589, Saudi Arabia; Tsinghua University, CHINA

## Abstract

This work deals with the flow and heat transfer in upper-convected Maxwell fluid above an exponentially stretching surface. Cattaneo-Christov heat flux model is employed for the formulation of the energy equation. This model can predict the effects of thermal relaxation time on the boundary layer. Similarity approach is utilized to normalize the governing boundary layer equations. Local similarity solutions are achieved by shooting approach together with fourth-fifth-order Runge-Kutta integration technique and Newton’s method. Our computations reveal that fluid temperature has inverse relationship with the thermal relaxation time. Further the fluid velocity is a decreasing function of the fluid relaxation time. A comparison of Fourier’s law and the Cattaneo-Christov’s law is also presented. Present attempt even in the case of Newtonian fluid is not yet available in the literature.

## Introduction

Heat transfer phenomenon is involved in wide ranging industrial and engineering processes including nuclear reactor cooling, space cooling, energy production, biomedical applications such as magnetic drug targeting, heat conduction in tissues etc. and many others. Heat conduction law proposed by Fourier [1] has been the basis to predict the heat transfer behavior in diverse practical situations. One of the major shortcomings of this model is that it produces a parabolic energy equation which means that an initial disturbance would instantly affect the system under consideration. To overcome this paradox, several modified versions of the Fourier’s law have been introduced (see for instance [2–4] and refs. therein). Cattaneo [5], in his famous article, amended the Fourier’s law with the inclusion of relaxation time for heat flux which is defined as the time required to establish steady heat conduction once a temperature gradient is imposed. A material invariant formulation of the Cattaneo’s model was presented by Christov [6] through the consideration of Oldroyd’s upper-convected derivative. Straughan [7] used Cattaneo-Christov model to investigate thermal convection in an incompressible flow. Structural stability and uniqueness of the Cattaneo-Christov equations were discussed by Ciarletta and Straughan [8]. Han et. al. [9] used Cattaneo-Christov law to explore the slip flow and heat transfer of viscoelastic fluid bounded by a stretching plate. Mustafa [10] computed both analytical and numerical solutions for rotating flow of Maxwell fluid with the consideration of Cattaneo-Christov heat flux.

The purpose of this paper is to study the boundary layer flow of upper-convected Maxwell (UCM) fluid induced by exponentially stretching sheet using Cattaneo-Christov heat flux model. Maxwell fluid is a popular viscoelastic fluid that can give the influence of fluid relaxation time. On the other hand, the study of viscous flow and heat transfer above stretching surfaces has been widely addressed research area due to its abundant applications in chemical and manufacturing processes including polymer extrusion, continuous casting of metals, extrusion of copper wires, die forging, paper production and several others. Several interesting boundary layer flow problems involving the stretching surfaces have been addressed in recent years [11–14]. The present work is motivated towards the influence of thermal relaxation time on the viscoelastic flow due to exponentially stretching surface. Some recent boundary layer flow problems involving Maxwell fluid can be found in refs. [15–27]. The equations are first simplified through boundary layer approximations and then local similarity solution is obtained by a numerical procedure. Emphasis is given to the role of relaxation time for heat flux on the boundary layers.

## Problem Formulation

Consider the steady two dimensional incompressible flow of upper-convected Maxwell (UCM) fluid over an elastic sheet located at *y* = 0. The sheet is stretched in its own plane with the velocity $U_{w}(x)=U_{0}e^{x/L}$. A variable surface temperature distribution of the form $T_{w}=T_{\infty }+T_{0}e^{Ax/2L}$ [15] is considered in which *T*_0_ denotes the heating/cooling reference temperature. This is reasonable since in extrusion process, the material properties and in particular the elasticity of the extruded sheet is being pulled out by a constant force. Invoking the boundary layer approximations, the equations governing the two-dimensional flow and heat transfer of incompressible UCM fluid are expressed as below:

$$\frac{\partial u}{\partial x}+\frac{\partial v}{\partial y}=0,$$ (1)

$$u\frac{\partial u}{\partial x}+v\frac{\partial u}{\partial y}+\lambda_{1}\left(u^{2}\frac{\partial^{2}u}{\partial x^{2}}+v^{2}\frac{\partial^{2}u}{\partial y^{2}}+2uv\frac{\partial^{2}u}{\partial x\partial y}\right)=\nu \frac{\partial^{2}u}{\partial y^{2}},$$ (2)

$$(\rho c_{p})\left(u\frac{\partial T}{\partial x}+v\frac{\partial T}{\partial y}\right)=-\nabla .q,$$ (3)

where *u* and *v* denote the velocity components along the *x*—and *y*—directions respectively, *v* is the kinematic viscosity, *λ*_1_ is the fluid relaxation time, *T* is the local fluid temperature and **q** is the heat flux which satisfies the following relationship [3].

$$q+\lambda_{2}\left(\frac{\partial q}{\partial t}+V.\nabla q-q.\nabla V+\left(\nabla .V\right)q\right)=-k\nabla T,$$ (4)

in which *λ*_2_ is the relaxation time for heat flux, **V** is the velocity vector and *k* is the thermal conductivity. Eliminating **q** from Eqs (3) and (4), we obtain the following (see Christov [3] and Han et al. [6])

$$u\frac{\partial T}{\partial x}+v\frac{\partial T}{\partial y}+\lambda_{2}\left[\left(u\frac{\partial u}{\partial x}+v\frac{\partial u}{\partial y}\right)\frac{\partial T}{\partial x}+\left(u\frac{\partial v}{\partial x}+v\frac{\partial v}{\partial y}\right)\frac{\partial T}{\partial y}+u^{2}\frac{\partial^{2}T}{\partial x^{2}}+v^{2}\frac{\partial^{2}T}{\partial y^{2}}+2uv\frac{\partial^{2}T}{\partial x\partial y}\right]=\alpha \frac{\partial^{2}T}{\partial y^{2}},$$ (5)

where *α* (= *k* / *ρc*_p_) is the thermal diffusivity. The boundary conditions are imposed as below:

$$\begin{array}{l} u=U_{w}(x)=U_{0}e^{x/L},\;v=0,\;T=T_{w}(x)=T_{\infty }+T_{0}e^{Ax/2L}\text{ at }y=0,\\ u\to 0,\;T\to T_{\infty }\text{ as }y\to \infty . \end{array}$$ (6)

Using the following similarity transformations [15]

$$\eta =\sqrt{\frac{U_{0}}{2\nu L}}e^{x/2L}y,\;u=U_{0}e^{x/L}f^{\prime },\;v=-\sqrt{\frac{\nu U_{0}}{2L}}e^{x/2L}\left(f+\eta f^{\prime }\right),\;\theta =\frac{T-T_{\infty }}{T_{w}-T_{\infty }},\;$$ (7)

Eq (1) is identically satisfied and Eqs (2)–(6) take the following forms

$$f^{\prime \prime \prime }-2{f^{\prime }}^{2}+ff^{\prime \prime }+\Lambda_{1}\left(3ff^{\prime }f^{\prime \prime }+\frac{\eta }{2}{f^{\prime }}^{2}f^{\prime \prime }-\frac{1}{2}f^{2}f^{\prime \prime \prime }-2{f^{\prime }}^{3}\right)=0,$$ (8)

$$\frac{1}{Pr}\theta^{\prime \prime }+f\theta^{\prime }-Af^{\prime }\theta +\;\frac{\Lambda_{2}}{2}\left[A\;ff^{\prime \prime }\theta -A\left(2+A\right)f^{\prime 2}\theta +\left(1+2A\right)ff^{\prime }\theta^{\prime }-f^{2}\theta^{\prime \prime }\right]=0,$$ (9)

$$\begin{array}{l} f(0)=0,\;f^{\prime }(0)=1,\;\theta (0)=1,\\ \;f^{\prime }(\infty )\to 0,\;\theta (\infty )\to 0, \end{array}$$ (10)

where $\Lambda_{1}=\lambda_{1}U_{0}e^{x/L}/L$ is the non-dimensional fluid relaxation time, $\Lambda_{2}=\lambda_{2}U_{0}e^{x/L}/L$ is the non-dimensional thermal relaxation time and *Pr* = *v* / *α* is the Prandtl number.

It is important to point out through the Eqs (8)–(10) that when Λ_1_ = 0, the case of Newtonian fluid is obtained. Further Λ_2_ = 0 corresponds to the case of classical Fourier’s heat conduction law.

## 1. Numerical method

We employ the shooting method with fifth order Runge-Kutta procedure for the numerical solution of the present problem. First of all we reduce the Eqs (8) and (9) and boundary conditions (10) into a system of 1^st^ order ODEs by making a substitution (*x*_1_, *x*_2_, *x*_3_, *x*_4_, *x*_5_) = (*f*, *f*’, *f*”, *θ*, *θ*’). This yields the following:

$$\left(\begin{array}{c} {x^{\prime }}_{1}\\ {x^{\prime }}_{2}\\ {x^{\prime }}_{3}\\ {x^{\prime }}_{4}\\ {x^{\prime }}_{5} \end{array}\right)=\left(\begin{array}{c} x_{2}\\ x_{3}\\ \left(2x_{2}{}^{2}-x_{1}x_{3}-\Lambda_{1}\left(3x_{1}x_{2}x_{3}+\frac{\eta }{2}x_{2}{}^{2}x_{3}-2x_{2}{}^{3}\right)\right)/\left(1-\frac{\Lambda_{1}}{2}x_{1}{}^{2}\right)\\ x_{5}\\ \left(-x_{1}x_{5}+Ax_{2}x_{4}-\frac{\Lambda_{2}}{2}\left(Ax_{1}x_{3}x_{4}-A\left(2+A\right)x_{2}{}^{2}x_{4}+\left(1+2A\right)x_{1}x_{2}x_{5}\right)\right)/\left(\frac{1}{\text{Pr}}-\frac{\Lambda_{2}}{2}x_{1}{}^{2}\right) \end{array}\right),$$ (11)

$$\left(\begin{array}{c} x_{1}\left(0\right)\\ x_{2}\left(0\right)\\ x_{3}\left(0\right)\\ x_{4}\left(0\right)\\ x_{5}\left(0\right) \end{array}\right)=\left(\begin{array}{c} 0\\ 1\\ f^{\prime \prime }(0)\\ 1\\ \theta^{\prime }(0) \end{array}\right),$$ (12)

Eq (11) subject to the initial conditions Eq (12) are integrated numerically using fifth-order Runge Kutta method through suitable choice of the unknown initial conditions *u*1 = *f*”(0) and *u*_2_ = *θ*’(0). The values of these conditions are then iteratively estimated through Newton’s method such that solutions satisfy the boundary conditions at infinity (given in Eq (10)) with the error less than 10^−5^.

## 2. Results and discussion

Physical interpretation to the behavior of the embedded parameters is assigned in this section. In Table 1 we presented the numerical values of wall temperature gradient for different values of embedded parameters. We notice that *θ*’(0) is directly proportional to the dimensionless relaxation time Λ_1_. However it appears to decrease upon increasing the fluid relaxation time. Notably, the value of *θ*’(0) is negative when *A* = −1.5 revealing the reverse flow near the wall which will be explained later. There is a significant growth in the wall temperature gradient *θ*’(0). When *A* is incremented. When *A* enlarges, this leads to larger a surface temperature and hence larger heat transfer rate from the sheet.

**Table 1:** Computational results of wall temperature gradient *θ*’(0) for different values of parameters.

*Pr*	Λ_1_	Λ_2_	*θ*’(0)		
			*A* = −1.5	*A* = 0	*A* = 1.5
0.7	0.5	0	0.235311	-0.395729	-0.84128
		0.5	0.378077	-0.431201	-1.14357
		1	0.535309	-0.47125	-1.42710
	0	0.5	0.440075	-0.474096	-1.22415
	0.5		0.378077	-0.431201	-1.14357
	1		0.334957	-0.402024	-1.08247
1	0.5	0	0.333441	-0.512599	-1.06969
		0.5	0.532307	-0.570367	-1.46365
		1	0.755562	-0.635466	-1.82605
	0	0.5	0.608711	-0.622927	-1.55096
	0.5		0.532307	-0.570367	-1.46365
	1		0.480170	-0.532685	-1.39552

**Fig 1:**
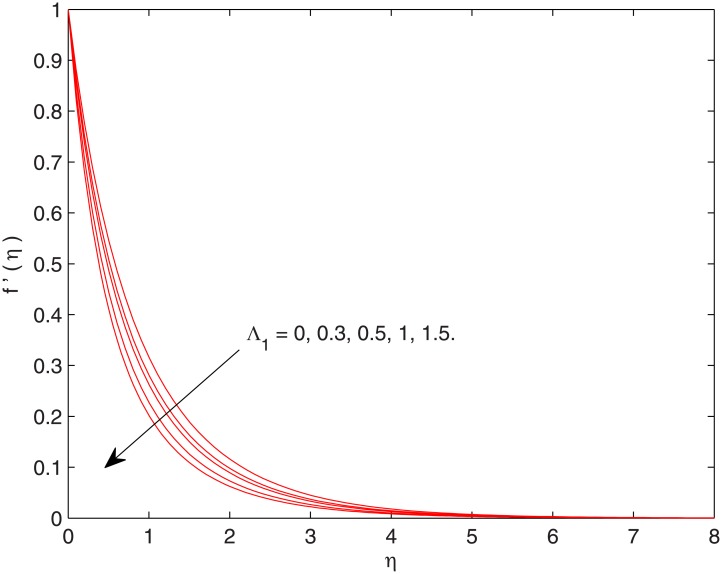
Effect of Λ_1_ on *f*’(*η*).

Fig 1 illustrates the effects of non-dimensional fluid relaxation time on the hydrodynamic boundary layer. An increase in Λ_1_ may be regarded as increase in fluid viscosity. This increased viscosity opposes the fluid motion and consequently the velocity decreases. It is also clear that velocity profiles are tilted towards the stretching wall when Λ_1_ is increased which means that boundary layer thickness is an increasing function of Λ_1_. The obtained results are in accordance with the results of Han et al. [6] in which linearly stretching sheet was considered.

**Fig 2:**
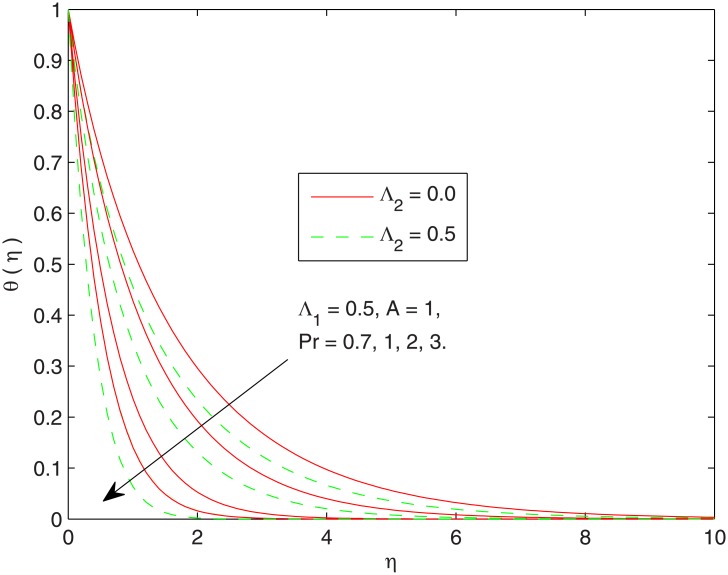
Effect of *Pr* on *θ*(*η*).

Fig 2 portrays the behavior of Prandtl number *Pr* on the thermal boundary layer with and without the consideration of thermal relaxation time. The behavior of *Pr* on *θ* is qualitatively similar in both the cases i.e. the temperature and thermal boundary layer thickness both are found to decrease upon increasing *Pr*. Notably the variation in temperature *θ* is similar in magnitude in both Fourier and Cattaneo-Christov heat flux models. Physically, the Prandtl number *Pr* is inversely related with the thermal diffusivity *α*. As *Pr* enlarges, one anticipates less thermal effect to penetrate into the fluid. Due to this reason the thermal boundary layer becomes thinner when *Pr* is increased. The thinner thermal boundary layer leads to a steeper temperature profile indicating larger wall slope of temperature function.

**Fig 3:**
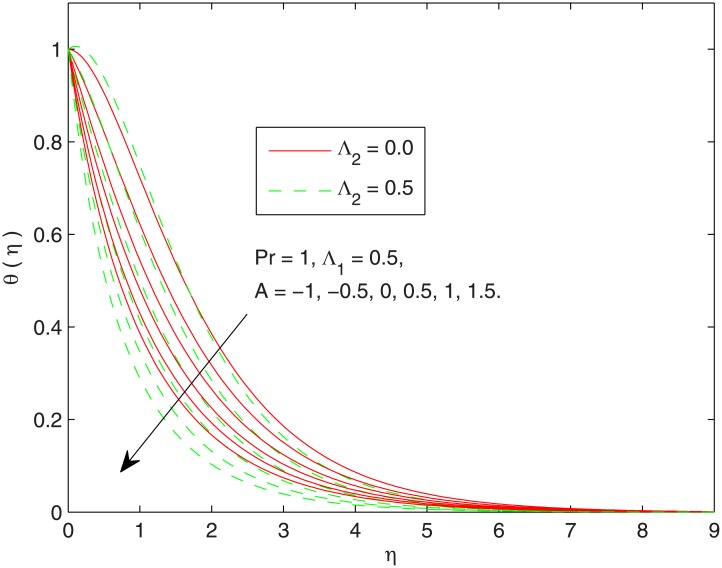
Effect of *A* on *θ*(*η*).

In Fig 3 the impact of temperature exponent *A* on the temperature profile is sketched. This Figdepicts an interesting phenomenon of “Sparrow-Gregg hill (SGH)” for negative temperature exponent *A* in which temperature *θ* first approaches to a maximum and then exponentially descends to zero when *η* is increased. This means that for some negative *A*, one expects reverse heat flow in the vicinity of the sheet. This result is consistent with the findings of Magyari and Keller [7] for the Fourier heat conduction law. With an increase in positive/negative temperature exponent parameter *A*, we observe a sharp growth in wall slope of temperature function.

**Fig 4:**
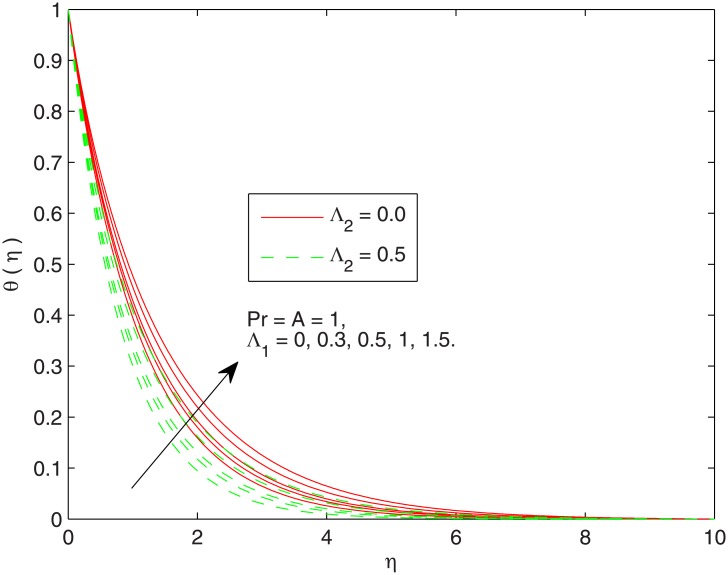
Effect of Λ_1_ on *θ*(*η*).

**Fig 5:**
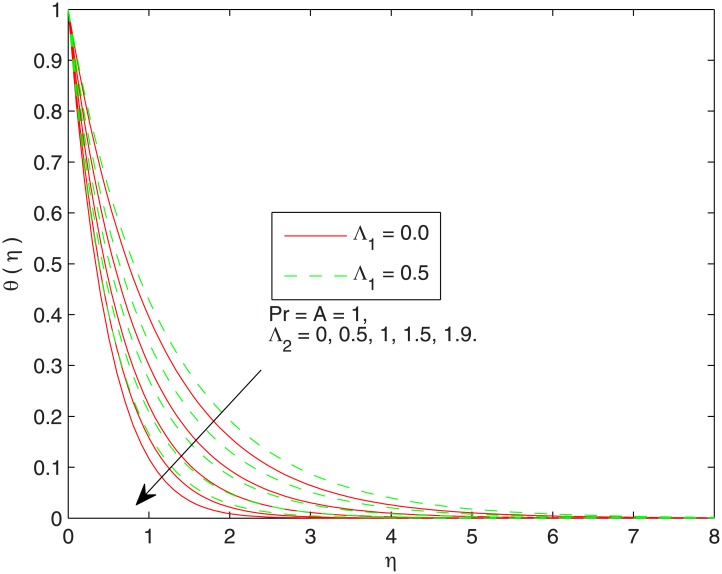
Effect of Λ_2_ on *θ*(*η*).

Fig 4 displays the influence of Λ_1_ on thermal boundary layer. Stronger viscous force associated with the larger Λ_1_ resists the flow and enhances the temperature. This leads to the conclusion that temperature in viscoelastic fluid is greater than the viscous fluid. In Fig 5 the effect of non-dimensional thermal relaxation time Λ_2_. on the temperature distribution is sketched. We observe that temperature *θ* has inverse relationship with the thermal relaxation time. We also notice that temperature *θ* approaches the free stream condition at shorter distances above the sheet for bigger Λ_2_. Notably, the variation in temperature *θ* with thermal relaxation time is of similar magnitude in Newtonian and Maxwell fluids.

**Fig 6:**
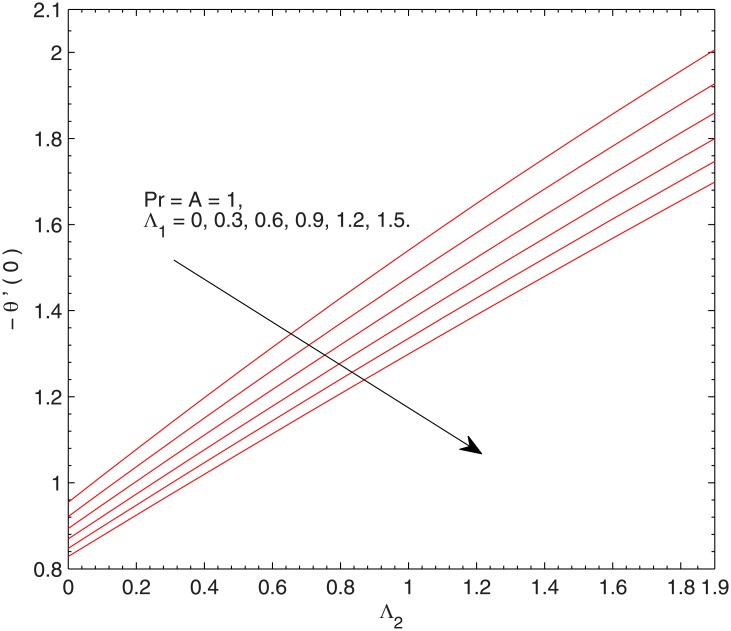
Effect of Λ_1_ and Λ_2_ on–*θ*’(0).

**Fig 7:**
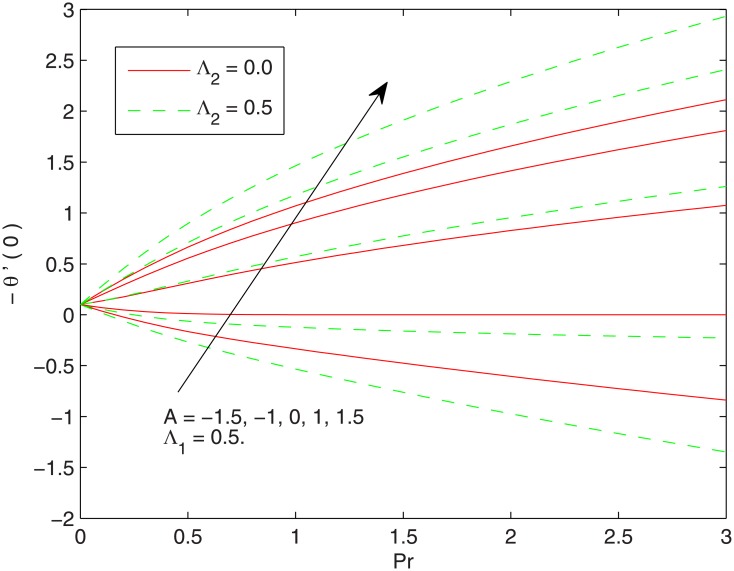
Effect of *Pr*, *A* and Λ_2_ on–*θ*’(0).

Fig 6 presents the wall temperature gradient as a function of relaxation time Λ_2_ at different values of Λ_1_. *θ*’(0) linearly increases with an increment in Λ_2_ whereas it appears to decrease when Λ_1_ is increased. Fig 7 plots *θ*’(0) against the Prandtl number with the variations in Λ_1_ and Λ_2_. This Fig is complementing the numerical results of *θ*’(0) given in Table 1. The profiles of *θ*’(0) are nearly a straight line revealing that heat transfer rate grows linearly when *Pr* is augmented. We observe that *θ*’(0) approaches to zero for vanishing Prandtl number.

## 3. Concluding remarks

Cattaneo-Christov heat flux model is used to describe the heat transfer in viscoelastic flow induced by an exponentially stretching sheet. The major points of this study may be summarized as under:

1. Hydrodynamic boundary layer is thinner in viscoelastic fluid when compared with the viscous fluid.
2. Temperature and thermal boundary layer thickness are decreasing functions of relaxation time Λ_2_.
3. Interesting Sparrow-Gregg Hills (SGH) for the temperature distribution exist for negative temperature exponent *A*.
4. The behaviors of parameters in Cattaneo-Christov model are qualitatively similar to those in Fourier’s heat conduction law.
5. The present consideration for the Newtonian fluid case can be recovered by choosing Λ_1_ = 0.
